# Methyl 2-(4-acetamido­benzene­sulfon­amido)­benzoate

**DOI:** 10.1107/S1600536811009500

**Published:** 2011-03-19

**Authors:** Islam Ullah Khan, Shahzad Sharif, Salamat Ali, Waqar Ahmad, Edward R. T. Tiekink

**Affiliations:** a Materials Chemistry Laboratory, Department of Chemistry, Government College University, Lahore 54000, Pakistan; b Department of Physics, Government College University, Lahore 54000, Pakistan; c Department of Chemistry, University of Malaya, 50603 Kuala Lumpur, Malaysia

## Abstract

The mol­ecule of the title compound, C_16_H_16_N_2_O_5_S, has the shape of the letter V but with a small twist; the dihedral angle formed between the benzene rings is 79.66 (9)°. The presence of an intra­molecular N—H⋯O hydrogen bond, leading to an *S*(6) ring, correlates with the near coplanarity of the carboxyl­ate ester group with the benzene ring to which it is connected. The acetamide residue is slightly twisted out of the plane of its benzene ring [C—C—N—C = 13.1 (3)°]. In the crystal, supra­molecular chains along the *a* axis are mediated by N—H⋯O hydrogen bonds. These are connected into layers *via* C—H⋯O inter­actions.

## Related literature

For background to the pharmacological uses of sulfonamides, see: Korolkovas (1988 ▶); Mandell & Sande (1992 ▶). For related structures, see: Sharif *et al.* (2010 ▶); Khan *et al.* (2010 ▶).
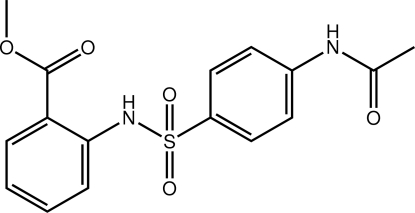


## Experimental

### 

#### Crystal data

C_16_H_16_N_2_O_5_S
*M* *_r_* = 348.37
Triclinic, 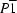

*a* = 8.2835 (2) Å
*b* = 9.3722 (3) Å
*c* = 10.8299 (3) Å
α = 85.537 (1)°
β = 88.614 (1)°
γ = 72.203 (1)°
*V* = 798.11 (4) Å^3^
*Z* = 2
Mo *K*α radiation
μ = 0.23 mm^−1^
*T* = 293 K
0.20 × 0.18 × 0.09 mm

#### Data collection

Bruker APEXII CCD diffractometer
13593 measured reflections
3604 independent reflections
3122 reflections with *I* > 2σ(*I*)
*R*_int_ = 0.024

#### Refinement

*R*[*F* ^2^ > 2σ(*F* ^2^)] = 0.038
*wR*(*F* ^2^) = 0.113
*S* = 1.07
3604 reflections
225 parameters
2 restraints
H atoms treated by a mixture of independent and constrained refinement
Δρ_max_ = 0.26 e Å^−3^
Δρ_min_ = −0.27 e Å^−3^

### 

Data collection: *APEX2* (Bruker, 2007 ▶); cell refinement: *SAINT* (Bruker, 2007 ▶); data reduction: *SAINT*; program(s) used to solve structure: *SHELXS97* (Sheldrick, 2008 ▶); program(s) used to refine structure: *SHELXL97* (Sheldrick, 2008 ▶); molecular graphics: *ORTEP-3* (Farrugia, 1997 ▶) and *DIAMOND* (Brandenburg, 2006 ▶); software used to prepare material for publication: *publCIF* (Westrip, 2010 ▶).

## Supplementary Material

Crystal structure: contains datablocks global, I. DOI: 10.1107/S1600536811009500/hb5817sup1.cif

Structure factors: contains datablocks I. DOI: 10.1107/S1600536811009500/hb5817Isup2.hkl

Additional supplementary materials:  crystallographic information; 3D view; checkCIF report

## Figures and Tables

**Table 1:** Hydrogen-bond geometry (Å, °)

*D*—H⋯*A*	*D*—H	H⋯*A*	*D*⋯*A*	*D*—H⋯*A*
N1—H1n⋯O3	0.86 (1)	1.90 (2)	2.6266 (18)	141 (2)
N2—H2n⋯O2^i^	0.86 (2)	2.31 (2)	3.0888 (19)	151 (2)
C10—H10⋯O1^ii^	0.93	2.57	3.330 (2)	140

Symmetry codes: (i) 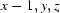; (ii) 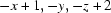.

**Fig. 1.**
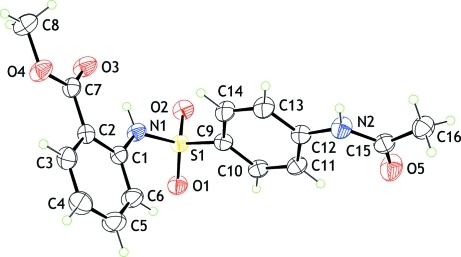
The molecular structure of (I) showing displacement ellipsoids at the 35% probability level.

**Fig. 2.**
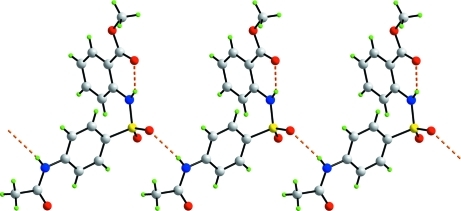
A view of the linear supramolecular chain along the a axis in (I), sustained by N—H···O interactions. These and the intramolecular N—H···O hydrogen bonds which close six-membered rings are shown as orange dashed lines.

**Fig. 3.**
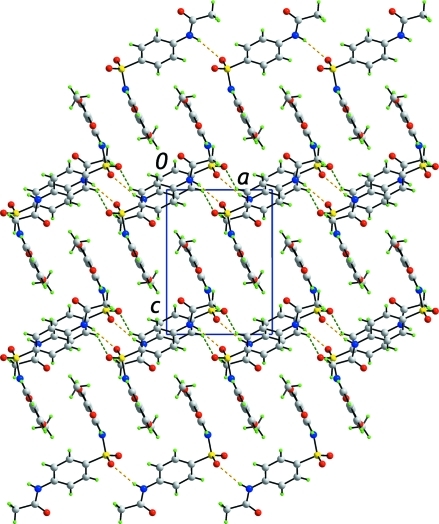
A view in projection down the b axis of the unit-cell contents for (I). The N—H···O hydrogen bonds and C—H···O contacts are shown as orange and green dashed lines, respectively.

## supplementary crystallographic information

### Comment

As part of on-going structural studies of sulfonamides (Sharif *et al.*, 2010; Khan *et al.*, 2010), the crystal structure of the title compound, (I), is described. Interest in these derivatives relate to their wide use in the treatment of certain infections caused by Gram-positive and Gram-negative microorganisms, some fungi, and certain protozoa (Korolkovas, 1988; Mandell & Sande, 1992).

The molecule of (I), Fig. 1, has an approximate V-shape with the dihedral angle formed between the benzene rings being 79.66 (9) °. The carboxylate ester substituent is co-planar with the benzene ring to which it is connected [the C1—C2—C7—O3 and torsion angle is 2.9 (2) °] but the acetamide residue is slightly twisted out of the plane [C11—C12—N2—C15 = 13.1 (3) °]. The planarity observed for the carboxylate ester group is readily rationalized in terms of an intramolecular N—H···O hydrogen bond, Table 1, which seals a six-membered ring. The most prominent intermolecular contact in the crystal structure is also of the type N—H···O, Table 1, and this serves to link molecules into a linear supramolecular chain along the *a* axis, Fig. 3. Chains are linked into layers in the *ab* plane *via* C—H···O contacts, Table 1, and these stack along the *c* axis *via* inter-digitation of the benzoate ester groups; there is no evidence for significant π-interactions between these, however.

### Experimental

To methyl anthranilate (260 µl, 2 mmol) in water (10 ml) was added *p*-toluene sulfonyl chloride (380 mg, 2 mmol). With stirring at room temperature, the pH of the solution was maintained with 3% Na_2_CO_3_. The progress of the reaction was monitored by TLC. On completion of the reaction, the pH was adjusted to 3 with 3 N HCl. The white precipitates that formed were filtered, washed with distilled water and crystallized from methanol to yield colourless blocks of the title compound.

### Refinement

The C-bound H atoms were geometrically placed (C–H = 0.93–0.96 Å) and refined as riding with *U_iso_*(H) = 1.2–1.5*U_eq_*(C). The N-bound H atoms were refined with the distance restraint N–H = 0.86±0.01 Å, and with *U_iso_*(H) = 1.2*U_eq_*(N). Several low-angle reflections, *i.e*. (1 0 1), (0 1 0), (0 0 1) and (1 1 0), evidently effected by the beam-stop, were omitted from the final refinement.

### Crystal data

**Table:**

C_16_H_16_N_2_O_5_S	*Z* = 2
*M**_r_* = 348.37	*F*(000) = 364
Triclinic, *P*1	*D*_x_ = 1.450 Mg m^−^^3^
Hall symbol: -P 1	Mo *K*α radiation, λ = 0.71073 Å
*a* = 8.2835 (2) Å	Cell parameters from 7451 reflections
*b* = 9.3722 (3) Å	θ = 2.3–28.2°
*c* = 10.8299 (3) Å	µ = 0.23 mm^−^^1^
α = 85.537 (1)°	*T* = 293 K
β = 88.614 (1)°	Block, colourless
γ = 72.203 (1)°	0.20 × 0.18 × 0.09 mm
*V* = 798.11 (4) Å^3^	

### Data collection

**Table:**

Bruker APEXII CCD diffractometer	3122 reflections with *I* > 2σ(*I*)
Radiation source: fine-focus sealed tube	*R*_int_ = 0.024
graphite	θ_max_ = 27.5°, θ_min_ = 3.4°
φ and ω scans	*h* = −10→10
13593 measured reflections	*k* = −12→12
3604 independent reflections	*l* = −14→12

### Refinement

**Table:**

Refinement on *F*^2^	Primary atom site location: structure-invariant direct methods
Least-squares matrix: full	Secondary atom site location: difference Fourier map
*R*[*F*^2^ > 2σ(*F*^2^)] = 0.038	Hydrogen site location: inferred from neighbouring sites
*wR*(*F*^2^) = 0.113	H atoms treated by a mixture of independent and constrained refinement
*S* = 1.07	*w* = 1/[σ^2^(*F*_o_^2^) + (0.0578*P*)^2^ + 0.1747*P*] where *P* = (*F*_o_^2^ + 2*F*_c_^2^)/3
3604 reflections	(Δ/σ)_max_ = 0.001
225 parameters	Δρ_max_ = 0.26 e Å^−^^3^
2 restraints	Δρ_min_ = −0.27 e Å^−^^3^

### Special details

**Table:**

Geometry. All s.u.'s (except the s.u. in the dihedral angle between two l.s. planes) are estimated using the full covariance matrix. The cell s.u.'s are taken into account individually in the estimation of s.u.'s in distances, angles and torsion angles; correlations between s.u.'s in cell parameters are only used when they are defined by crystal symmetry. An approximate (isotropic) treatment of cell s.u.'s is used for estimating s.u.'s involving l.s. planes.
Refinement. Refinement of *F*^2^ against ALL reflections. The weighted *R*-factor *wR* and goodness of fit *S* are based on *F*^2^, conventional *R*-factors *R* are based on *F*, with *F* set to zero for negative *F*^2^. The threshold expression of *F*^2^ > 2σ(*F*^2^) is used only for calculating *R*-factors(gt) *etc*. and is not relevant to the choice of reflections for refinement. *R*-factors based on *F*^2^ are statistically about twice as large as those based on *F*, and *R*- factors based on ALL data will be even larger.

### Fractional atomic coordinates and isotropic or equivalent isotropic displacement parameters (Å^2^)

**Table:**

	*x*	*y*	*z*	*U*_iso_*/*U*_eq_	
S1	0.42614 (5)	0.28976 (4)	0.83592 (3)	0.04378 (14)	
O1	0.54431 (14)	0.14234 (13)	0.83938 (11)	0.0562 (3)	
O2	0.47186 (15)	0.40243 (13)	0.89646 (11)	0.0544 (3)	
O3	0.30254 (19)	0.61672 (14)	0.55728 (12)	0.0659 (4)	
O4	0.17057 (17)	0.62717 (14)	0.37946 (11)	0.0620 (3)	
O5	−0.13574 (18)	0.09502 (17)	1.23293 (14)	0.0808 (5)	
N1	0.38913 (18)	0.36108 (15)	0.69410 (12)	0.0477 (3)	
H1N	0.378 (2)	0.4554 (11)	0.6809 (18)	0.057*	
N2	−0.21841 (17)	0.26393 (16)	1.06806 (13)	0.0534 (3)	
H2N	−0.3050 (18)	0.3309 (17)	1.0351 (17)	0.064*	
C1	0.32524 (18)	0.30069 (16)	0.59818 (13)	0.0423 (3)	
C2	0.25586 (17)	0.39431 (17)	0.49240 (13)	0.0414 (3)	
C3	0.1942 (2)	0.3327 (2)	0.39815 (15)	0.0544 (4)	
H3	0.1487	0.3934	0.3276	0.065*	
C4	0.1987 (3)	0.1845 (2)	0.40639 (18)	0.0653 (5)	
H4	0.1569	0.1453	0.3424	0.078*	
C5	0.2660 (3)	0.0955 (2)	0.51059 (19)	0.0678 (5)	
H5	0.2690	−0.0048	0.5172	0.081*	
C6	0.3289 (3)	0.15192 (19)	0.60507 (17)	0.0608 (5)	
H6	0.3746	0.0893	0.6747	0.073*	
C7	0.24751 (19)	0.55468 (18)	0.48195 (14)	0.0456 (3)	
C8	0.1511 (3)	0.7859 (2)	0.3623 (2)	0.0703 (5)	
H8A	0.0954	0.8348	0.4332	0.105*	
H8B	0.0843	0.8281	0.2896	0.105*	
H8C	0.2607	0.8003	0.3526	0.105*	
C9	0.23368 (18)	0.27393 (16)	0.89638 (13)	0.0423 (3)	
C10	0.2359 (2)	0.15891 (17)	0.98514 (15)	0.0482 (4)	
H10	0.3379	0.0856	1.0056	0.058*	
C11	0.0888 (2)	0.15200 (17)	1.04354 (15)	0.0497 (4)	
H11	0.0915	0.0750	1.1038	0.060*	
C12	−0.06421 (19)	0.26073 (17)	1.01202 (14)	0.0445 (3)	
C13	−0.0658 (2)	0.37314 (19)	0.91947 (16)	0.0552 (4)	
H13	−0.1682	0.4438	0.8958	0.066*	
C14	0.0813 (2)	0.38105 (19)	0.86290 (15)	0.0531 (4)	
H14	0.0790	0.4578	0.8024	0.064*	
C15	−0.2473 (2)	0.18603 (18)	1.17324 (16)	0.0511 (4)	
C16	−0.4303 (2)	0.2227 (2)	1.20973 (19)	0.0614 (4)	
H16A	−0.4755	0.1470	1.1837	0.092*	
H16B	−0.4925	0.3188	1.1708	0.092*	
H16C	−0.4401	0.2258	1.2981	0.092*	

### Atomic displacement parameters (Å^2^)

**Table:**

	*U*^11^	*U*^22^	*U*^33^	*U*^12^	*U*^13^	*U*^23^
S1	0.0411 (2)	0.0446 (2)	0.0414 (2)	−0.00745 (15)	−0.01326 (14)	0.00368 (15)
O1	0.0458 (6)	0.0516 (6)	0.0599 (7)	−0.0004 (5)	−0.0059 (5)	0.0083 (5)
O2	0.0539 (6)	0.0594 (7)	0.0508 (6)	−0.0184 (5)	−0.0218 (5)	0.0008 (5)
O3	0.0896 (9)	0.0515 (7)	0.0588 (7)	−0.0249 (7)	−0.0269 (7)	0.0045 (6)
O4	0.0771 (8)	0.0539 (7)	0.0532 (7)	−0.0195 (6)	−0.0222 (6)	0.0127 (5)
O5	0.0625 (8)	0.0858 (10)	0.0740 (9)	−0.0021 (7)	−0.0046 (7)	0.0327 (8)
N1	0.0589 (8)	0.0433 (7)	0.0412 (6)	−0.0166 (6)	−0.0140 (6)	0.0035 (5)
N2	0.0406 (7)	0.0577 (8)	0.0547 (8)	−0.0074 (6)	−0.0118 (6)	0.0118 (6)
C1	0.0414 (7)	0.0455 (8)	0.0390 (7)	−0.0120 (6)	−0.0037 (6)	−0.0021 (6)
C2	0.0358 (7)	0.0489 (8)	0.0385 (7)	−0.0118 (6)	−0.0021 (5)	−0.0012 (6)
C3	0.0578 (9)	0.0642 (10)	0.0419 (8)	−0.0195 (8)	−0.0102 (7)	−0.0015 (7)
C4	0.0777 (12)	0.0718 (12)	0.0552 (10)	−0.0325 (10)	−0.0098 (9)	−0.0160 (9)
C5	0.0925 (14)	0.0533 (10)	0.0649 (11)	−0.0313 (10)	−0.0081 (10)	−0.0085 (8)
C6	0.0831 (13)	0.0480 (9)	0.0519 (9)	−0.0214 (9)	−0.0140 (9)	0.0021 (7)
C7	0.0423 (7)	0.0508 (8)	0.0412 (7)	−0.0114 (6)	−0.0055 (6)	0.0025 (6)
C8	0.0795 (13)	0.0520 (10)	0.0739 (12)	−0.0156 (9)	−0.0132 (10)	0.0151 (9)
C9	0.0414 (7)	0.0416 (7)	0.0395 (7)	−0.0060 (6)	−0.0121 (6)	−0.0003 (6)
C10	0.0416 (8)	0.0423 (8)	0.0523 (8)	−0.0018 (6)	−0.0128 (6)	0.0067 (6)
C11	0.0490 (8)	0.0429 (8)	0.0513 (8)	−0.0075 (7)	−0.0112 (7)	0.0086 (6)
C12	0.0421 (7)	0.0464 (8)	0.0421 (7)	−0.0091 (6)	−0.0121 (6)	0.0007 (6)
C13	0.0422 (8)	0.0577 (9)	0.0530 (9)	0.0001 (7)	−0.0135 (7)	0.0145 (7)
C14	0.0466 (8)	0.0538 (9)	0.0482 (8)	−0.0035 (7)	−0.0124 (7)	0.0157 (7)
C15	0.0518 (9)	0.0476 (8)	0.0523 (9)	−0.0137 (7)	−0.0066 (7)	0.0011 (7)
C16	0.0546 (10)	0.0624 (11)	0.0671 (11)	−0.0200 (8)	−0.0032 (8)	0.0051 (8)

### Geometric parameters (Å, °)

**Table:**

S1—O1	1.4267 (11)	C5—C6	1.370 (3)
S1—O2	1.4328 (12)	C5—H5	0.9300
S1—N1	1.6241 (13)	C6—H6	0.9300
S1—C9	1.7525 (16)	C8—H8A	0.9600
O3—C7	1.2104 (19)	C8—H8B	0.9600
O4—C7	1.3237 (18)	C8—H8C	0.9600
O4—C8	1.444 (2)	C9—C10	1.383 (2)
O5—C15	1.208 (2)	C9—C14	1.387 (2)
N1—C1	1.4074 (19)	C10—C11	1.377 (2)
N1—H1n	0.861 (11)	C10—H10	0.9300
N2—C15	1.361 (2)	C11—C12	1.392 (2)
N2—C12	1.394 (2)	C11—H11	0.9300
N2—H2n	0.859 (16)	C12—C13	1.394 (2)
C1—C6	1.381 (2)	C13—C14	1.370 (2)
C1—C2	1.407 (2)	C13—H13	0.9300
C2—C3	1.391 (2)	C14—H14	0.9300
C2—C7	1.479 (2)	C15—C16	1.499 (2)
C3—C4	1.373 (3)	C16—H16A	0.9600
C3—H3	0.9300	C16—H16B	0.9600
C4—C5	1.371 (3)	C16—H16C	0.9600
C4—H4	0.9300		
			
O1—S1—O2	117.99 (7)	O4—C8—H8A	109.5
O1—S1—N1	110.76 (7)	O4—C8—H8B	109.5
O2—S1—N1	103.67 (7)	H8A—C8—H8B	109.5
O1—S1—C9	107.67 (7)	O4—C8—H8C	109.5
O2—S1—C9	109.45 (7)	H8A—C8—H8C	109.5
N1—S1—C9	106.78 (7)	H8B—C8—H8C	109.5
C7—O4—C8	116.93 (14)	C10—C9—C14	119.92 (15)
C1—N1—S1	126.68 (11)	C10—C9—S1	119.16 (11)
C1—N1—H1N	113.9 (13)	C14—C9—S1	120.77 (12)
S1—N1—H1N	116.7 (13)	C11—C10—C9	120.63 (14)
C15—N2—C12	128.75 (13)	C11—C10—H10	119.7
C15—N2—H2N	116.5 (14)	C9—C10—H10	119.7
C12—N2—H2N	114.4 (14)	C10—C11—C12	119.75 (14)
C6—C1—C2	119.11 (14)	C10—C11—H11	120.1
C6—C1—N1	121.77 (14)	C12—C11—H11	120.1
C2—C1—N1	119.12 (13)	C11—C12—N2	123.53 (14)
C3—C2—C1	118.39 (14)	C11—C12—C13	119.09 (15)
C3—C2—C7	120.74 (14)	N2—C12—C13	117.38 (13)
C1—C2—C7	120.86 (13)	C14—C13—C12	120.98 (14)
C4—C3—C2	121.80 (16)	C14—C13—H13	119.5
C4—C3—H3	119.1	C12—C13—H13	119.5
C2—C3—H3	119.1	C13—C14—C9	119.58 (14)
C5—C4—C3	118.83 (17)	C13—C14—H14	120.2
C5—C4—H4	120.6	C9—C14—H14	120.2
C3—C4—H4	120.6	O5—C15—N2	123.33 (16)
C6—C5—C4	121.09 (17)	O5—C15—C16	122.38 (16)
C6—C5—H5	119.5	N2—C15—C16	114.29 (14)
C4—C5—H5	119.5	C15—C16—H16A	109.5
C5—C6—C1	120.77 (17)	C15—C16—H16B	109.5
C5—C6—H6	119.6	H16A—C16—H16B	109.5
C1—C6—H6	119.6	C15—C16—H16C	109.5
O3—C7—O4	122.11 (15)	H16A—C16—H16C	109.5
O3—C7—C2	125.32 (14)	H16B—C16—H16C	109.5
O4—C7—C2	112.57 (13)		
			
O1—S1—N1—C1	58.39 (15)	C1—C2—C7—O4	−176.68 (13)
O2—S1—N1—C1	−174.12 (13)	O1—S1—C9—C10	30.05 (15)
C9—S1—N1—C1	−58.57 (15)	O2—S1—C9—C10	−99.35 (14)
S1—N1—C1—C6	−17.9 (2)	N1—S1—C9—C10	149.04 (13)
S1—N1—C1—C2	162.13 (12)	O1—S1—C9—C14	−154.60 (13)
C6—C1—C2—C3	−0.3 (2)	O2—S1—C9—C14	75.99 (14)
N1—C1—C2—C3	179.62 (14)	N1—S1—C9—C14	−35.61 (15)
C6—C1—C2—C7	179.24 (15)	C14—C9—C10—C11	−1.9 (2)
N1—C1—C2—C7	−0.8 (2)	S1—C9—C10—C11	173.44 (12)
C1—C2—C3—C4	0.4 (2)	C9—C10—C11—C12	0.8 (2)
C7—C2—C3—C4	−179.21 (16)	C10—C11—C12—N2	−178.72 (15)
C2—C3—C4—C5	0.0 (3)	C10—C11—C12—C13	1.4 (2)
C3—C4—C5—C6	−0.4 (3)	C15—N2—C12—C11	13.1 (3)
C4—C5—C6—C1	0.4 (3)	C15—N2—C12—C13	−167.05 (17)
C2—C1—C6—C5	0.0 (3)	C11—C12—C13—C14	−2.5 (3)
N1—C1—C6—C5	180.00 (17)	N2—C12—C13—C14	177.67 (16)
C8—O4—C7—O3	−1.4 (2)	C12—C13—C14—C9	1.3 (3)
C8—O4—C7—C2	178.13 (15)	C10—C9—C14—C13	0.9 (3)
C3—C2—C7—O3	−177.58 (16)	S1—C9—C14—C13	−174.40 (13)
C1—C2—C7—O3	2.9 (2)	C12—N2—C15—O5	−2.5 (3)
C3—C2—C7—O4	2.9 (2)	C12—N2—C15—C16	177.36 (16)

### Hydrogen-bond geometry (Å, °)

**Table:**

*D*—H···*A*	*D*—H	H···*A*	*D*···*A*	*D*—H···*A*
N1—H1n···O3	0.861 (11)	1.902 (16)	2.6266 (18)	140.9 (17)
N2—H2n···O2^i^	0.859 (16)	2.311 (17)	3.0888 (19)	150.7 (15)
C10—H10···O1^ii^	0.93	2.57	3.330 (2)	140

Symmetry codes: (i) *x*−1, *y*, *z*; (ii) −*x*+1, −*y*, −*z*+2.
